# The Neuroticism Paradox: Direct and Indirect Effects on Work Performance

**DOI:** 10.1192/j.eurpsy.2026.10829

**Published:** 2026-07-31

**Authors:** N. Rmadi, R. Affes, N. Kotti, F. Dhouib, M. Hajjaji, K. Jmal Hammami

**Affiliations:** 1 Occupational medicine department, Hedi Chaker university hospital; 2 Family medecine department, Faculty of medecine of Sfax, Sfax, Tunisia

## Abstract

**Introduction:**

Neuroticism is traditionally viewed as a maladaptive trait associated with poorer occupational outcomes. However, its role may be more complex in high-stress environments such as residency training.

**Objectives:**

To explore both direct and indirect effects of neuroticism on job performance, with a focus on the mediating role of digital addiction among residents.

**Methods:**

A cross-sectional analysis of 429 Tunisian residents was conducted. Neuroticism was measured with the BFI-20, performance with IWPQ, smartphone addiction with the SAS-SV, and social media addiction with the BSMAS. Mediation models assessed direct and indirect effects.

**Results:**

Neuroticism was positively associated with overall job performance (β=0.268, p<0.001) and inversely correlated with counterproductive behaviors (r=–0.214, p<0.001). However, indirect pathways revealed detrimental effects through smartphone addiction (β=–0.161, p<0.001) and social media addiction (β=–0.253, p<0.001), both of which undermined performance.

**Conclusions:**

Neuroticism emerges as a paradoxical trait: it may foster vigilance and enhance performance directly, while simultaneously heightening susceptibility to digital addiction that diminishes its advantages. These findings highlight the need to account for both adaptive and maladaptive pathways when examining personality traits in occupational contexts.

**Disclosure of Interest:**

None Declared

